# Genome sequencing of multidrug resistant novel *Clostridium* sp. BL8 reveals its potential for pathogenicity

**DOI:** 10.1186/1757-4749-6-30

**Published:** 2014-07-18

**Authors:** Nachiket Prakash Marathe, Sudarshan Anand Shetty, Vikram B Lanjekar, Mandar Hemant Rasane, Dilip R Ranade, Yogesh S Shouche

**Affiliations:** 1 Microbial Culture Collection, National Centre for Cell Science, NCCS Complex, Ganeshkhind, Pune 411 007, Maharashtra, India; 2 Agharkar Research Institute, Gopal Ganesh Agarkar Road, Pune 411004, Maharashtra, India; 3 Current Address: Department of Infectious Diseases, Institute of Biomedicine, The Sahlgrenska Academy at the University of Gothenburg, Guldhedsgatan 10, SE-413 46, Göteborg, Sweden

**Keywords:** *Clostridium*, Virulence, Antibiotic resistance, Ion Torrent PGM^™^

## Abstract

**Background:**

The human gut microbiome is important for maintaining the health status of the host. Clostridia are key members of the human gut microbiome, carrying out several important functions in the gut environment. Hence understanding the role of different *Clostridium* species isolated from human gut is essential. The present study was aimed at investigating the role of novel *Clostridium* sp. isolate BL8 in human gut using genome sequencing as a tool.

**Findings:**

The genome analysis of *Clostridium* sp. BL8 showed the presence of several adaptive features like bile resistance, presence of sensory and regulatory systems, presence of oxidative stress managing systems and presence of membrane transport systems. The genome of *Clostridium* sp. BL8 consists of a wide variety of virulence factors like phospholipase C (alpha toxin), hemolysin, aureolysin and exfoliative toxin A, as well as adhesion factors, proteases, Type IV secretion system and antibiotic resistance genes. *In vitro* antibiotic sensitivity testing showed that *Clostridium* sp. BL8 was resistant to 11 different tested antibiotics belonging to 6 different classes. The cell cytotoxicity assay confirmed the cytotoxic effect of *Clostridium* sp. BL8 cells, which killed 40% of the Vero cells after 4 hrs of incubation.

**Conclusions:**

*Clostridium* sp. BL8 has adapted for survival in human gut environment, with presence of different adaptive features. The presence of several virulence factors and cell cytotoxic activity indicate that *Clostridium* sp. BL8 has a potential to cause infections in humans, however further *in vivo* studies are necessary to ascertain this fact.

## Background

The human gut is an intricate environment which harbors a large variety of microorganisms. The interaction between the host and the gut microbiome has an effect on the host health. The human gut microbiome is dominated by two bacterial phyla, *Firmicutes* and *Bacteroidetes*[1,2]. The *Firmicutes* population in the gut is dominated by the genus *Clostridium*. Clostridia start to colonize the human gut from the first month of life. *Clostridium* species in the gut play vital roles in degradation of food products, production of vitamins and short chain fatty acids, maintaining gut homeostasis and shaping the mucosal immune system. Recent studies have shown that Clostridia are reduced in abundance in inflammatory conditions such as inflammatory bowel disease (IBD), which leads to the dysbiosis further leading to inflammation [2]. Thus, Clostridia are key members of the human gut microbiome.

Although this is true, some pathogenic *Clostridium* species like *C. difficile*, *C. perfringens* and *C. tetani* have been described. These organisms cause fatal infections like colitis, gangrene and tetanus respectively [3-5]. Hence, understanding the role of *Clostridium* species isolated from the human gut is of paramount importance. In this study, we carried out genome sequencing of a novel *Clostridium* species (isolate BL8), isolated from the stool sample of a healthy Indian individual, in order to decipher the potential role of this organism in the human gut ecosystem. Our study demonstrates that *Clostridium* sp BL8 has a potential to cause infections in humans and lays basis for further studies to characterize this potential novel pathogen.

## Material and methods

### Genomic DNA extraction and 16S rRNA gene PCR and antibiotic sensitivity

Genomic DNA extraction and 16S rRNA gene sequencing was carried out as described earlier [6]. The antibiotic sensitivity of *Clostridium* sp. BL8 was carried out using antibiotic discs purchased from HiMedia laboratories, Mumbai following the CLSI guidelines [7].

### Genome sequencing and bioinformatic analysis

The genome sequencing using an Ion Torrent PGM™ and bioinformatic analysis was carried out using the methodology described by Shetty et al. [8].

### Cytotoxicity assay

The cell cytotoxicity assay was carried out by microculture tetrazolium (MTT) assay as described earlier [9]. Briefly: 96-well microplate was seeded with 100 μl medium containing 10,000 Vero cells in suspension. After 24 hr incubation and attachment, the cells were treated with 100 μl of 1 × 10^5^*Clostridium* sp. BL8 cell suspension or 100 μl cell free supernatant for 2 hrs and 4 hrs respectively, in triplicates. Cell suspension buffer and sterile culture medium were used as controls. Cell viability was determined by adding tetrazolium salt (5 mg/ml) (Sigma Aldrich, USA). After 3 hours of incubation at 37°C, media was removed and precipitated formazan was dissolve in 100 μl of DMSO. Absorbance was taken at 570 nm using an ELISA plate reader Spectra MAX250 (Molecular Devices, USA).

### Findings

### The isolate used in the study

**Figure 1:**
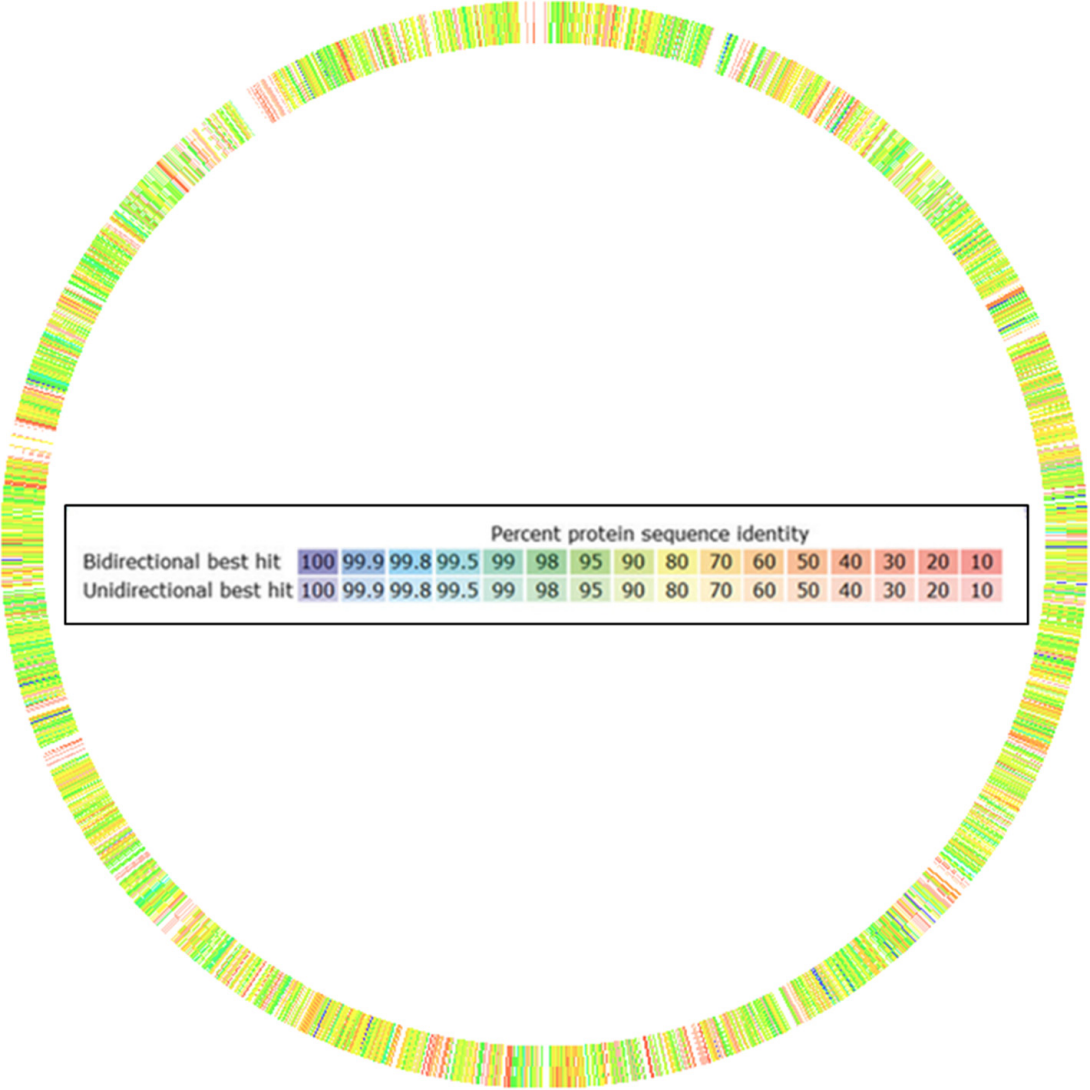
**Comparison of protein sequences of *Clostridium *sp. BL8 and *C. tunisiense *based on comparative genome analysis using SEED software.** The lines on circle indicate the alignment of predicted protein sequences from both the genomes and the color indicates the percent similarity between the predicted protein sequences. Genome sequence of *C. tunisiense* [GenBank: AMQH00000000] is used as a reference for alignment of the genome sequence of *Clostridium* sp. BL8.

The *Clostridium* sp. BL8 (CCUG 64195) has been reported in our previous study [6]. Based on 16S rRNA sequence [GenBank: JN093128] the closest validly published species are *Clostridium subterminale* and *Clostridium sulfidigenes* (97% sequence similarity in both the cases). This suggests that *Clostridium* sp. BL8 represents a novel species. *Clostridium* sp. BL8 shares 99% 16S rRNA sequence similarity with *C. tunisiense*, but *C. tunisiense* is not yet included in the list of standing nomenclature (http://www.bacterio.net). The comparison of genome sequences of BL8 and *C. tunisiense* [GenBank: AMQH00000000] suggested a very low sequence similarity between the genomes Figure 1. This further approves the fact that *Clostridium* sp. isolate BL8 represents a novel bacterial species belonging to the genus *Clostridium*.

### Genomic features of *Clostridium* sp. BL8

The assembled genome of *Clostridium* sp. BL8 was 4,776,227 bp with an average coverage of 37×. This Whole Genome Shotgun project has been deposited at DDBJ/EMBL/GenBank under the accession AUPA00000000. The list of all proteins annotated by RAST server is represented in Additional file 1: Table S1.

The genome of *Clostridium* sp. BL8 revealed the presence of adaptive features like bile resistance, presence of sensory and regulatory systems, presence of oxidative stress managing systems and presence of membrane transport systems. All these features are important for the survival of *Clostridium* sp. BL8 in the human gut [8].

### Resistance to antibiotics

The *in vitro* antibiotic sensitivity test demonstrated multi-drug resistance pattern of *Clostridium* sp. BL8, with resistance to 11 antibiotics belonging to 6 different classes. The genome sequencing revealed the presence of antibiotic resistance genes conferring resistance against these tested as well as other non tested antibiotics. The list of antibiotic resistance genes and tested antibiotics is represented in Table 1. MDR-type ABC transporters, drug/metabolite transporters (DMT), multidrug and toxin extrusion (MATE) family efflux pumps were also detected in the genome of BL8.

**Table 1:** **Antibiotic resistance genes detected in *Clostridium *** **sp. BL8 genome**

**Gene**	**Antibiotic class**	**Resistance tested against**
Aminoglycoside 6-adenylyltransferase	Aminoglycosides	Streptomycin, Amikacin
GCN5-related N-acetyltransferase	Aminoglycosides	Gentamicin, Kanamycin
Response regulator for bacitracin transporter BceR	Cyclic peptides	Colistin
bacitracin ABC transporter, ATP-binding protein	Cyclic peptides	Colistin
Metal-dependent beta-lactamase PhnP	Beta lactams	Penicillin-G
Beta-lactamase (EC 3.5.2.6)	Beta lactams	Ampicillin
Ribosome protection-type tetracycline resistance proteins	Tetracyclines	Tetracycline
Tetracycline efflux pump TetA	Tetracyclines	Tetracycline
Chloramphenicol acetyltransferase	Amphenicols	Chloramphenicol
Zwittermicin A resistance protein ZmaR	Aminopolyol	NT
Vancomycin B-type resistance protein VanW	Glycopeptides	Vancomycin
Quinolone resistance protein	Quinolones	NT
Macrolide-efflux protein	Macrolides	NT
Erythromycin esterase	Macrolides	NT
Erythromycin esterase type II	Macrolides	NT
Dihydrofolate reductase	Sulfonamides	Cotrimoxazole

Legend: NT- resistance not tested *in vitro*.

Horizontal gene transfer between pathogens and human commensals is an important factor for the dissemination of antibiotic resistance [10]. BL8 genome has mobile genetic elements like integrase and transposes such as IS1380-Spn, ISSod4, IS1294, IS3/IS911, IS110 and IS4. Type IV secretion system are important for transfer of DNA and secretion of proteins in bacteria [11]. BL8 genome encodes for type IV secretion system with presence of PilABC and PilMNO proteins and pilus regulator PilR. These systems would enable exchange of DNA between BL8 and other gut commensals.

### Genes coding for virulence factors

The virulence genes were categorized into two categories 1) toxins, 2) exoenzyme, adhesins and others. 1) Toxins and hemolysins: *C. perfringens* is known to produce alpha toxin (Phospholipase C) which is responsible for myonecrosis eventually causing gangrene [4]. Phospholipase C (EC 3.1.4.3) was detected in the genome of BL8 suggesting that it has a potential to cause muscle necrosis. The genes coding for hemolysin and hemolysin III were also detected in BL8 genome. All these genes are important virulence factors for *C. perfringens*[4]. Proteins similar to aureolysin and exfoliative toxin A of *Stahpyhlococcus* were also detected in BL8 genome. Exfoliative toxin A is known as ‘epidermolytic toxins’ which cleaves upper epidermal layer of skin, while aureolysin is a known complement inhibitor [12,13]. Both these genes are important for *S. aureus* pathogenesis.

2) Exoenzymes, adhensins and others: Secreted enzymes like proteases and serine proteases degrade host extracellular matrix allowing the entry of pathogens in the tissue, thus are important factors for virulence in bacterial pathogens. Secreted proteins like prepilin peptidase, HtrA family serine proteases, subtilisin-type proteinase, peptidase M23B were detected in BL8 genome. All these proteins play an important role in pathogenesis of various pathogens like *Legionella pneumophila*, *Streptococcus pyogenes*, *Streptococcus suis*, *Pseudomonas aeruginosa*, *Salmonella typhimurium*, *Campylobacter jejuni* and *Helicobacter pylori*[14-18].

Along with secreted proteins the genome of BL8 had myosin reactive antigen, which is reported to be important for virulence in *Shigella flexneri*[19]. BL8 genome also contains platelet-activating factor acetylhydrolase and iron ABC uptake transporter similar to anthrachelin. The platelet-activating factor acetylhydrolase is a proinflammatory enzyme required for virulence, while anthrachelin is essential for virulence of *Bacillus anthracis*[20,21]. Fibronectin/fibrinogen-binding protein, adhensins are important for adhesion and colonization of the pathogen on wound tissue and blood clots [5]. Genes coding for both these factors were present in the genome of BL8. To aid the regulations of these genes, several transcriptional regulators belonging to AraC, DeoR, GntR, LysR, MarR and TetR families were detected in the genome of BL8. All these regulators have been reported to be important in the regulation of several virulence genes in pathogens [22,23]. The presence of all these virulence genes and regulatory factors suggests that *Clostridium* sp. BL8 has a high potential for pathogenicity.

### Cytotoxicity assay

**Figure 2:**
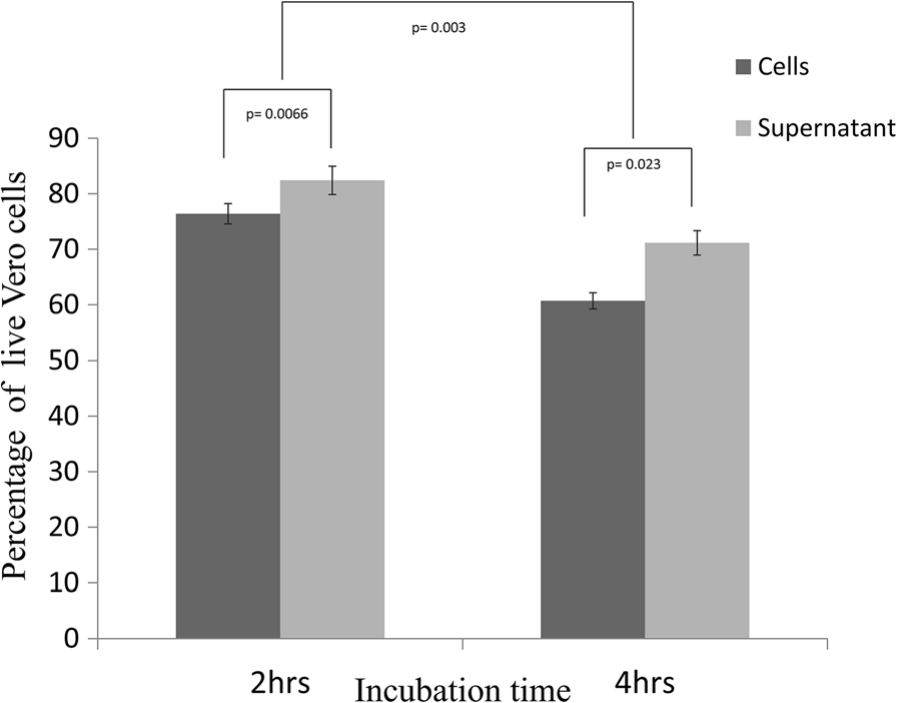
**The cytotoxic activity exhibited by *Clostridium *sp. BL8 cells and the cell free supernatant after 2 hours and 4 hours of incubation with the Vero cells.** The reading for controls (Vero cells without BL8 cell treatment) is considered as 100% cell viability. The error bars represent the standard deviations of three technical replicates.

Pathogens such as *C. perfringens* and *C. difficile* are known to have cytotoxic activity [24,25]. To investigate whether *Clostridium* sp. BL8 possesses any cytotoxic activity, we tested the *in vitro* cytotoxic effect of *Clostridium* sp. BL8 on Vero cells by MTT assay. Vero cells were used for the assay as this cell line has been recommended for the assay of *C. perfringens* enterotoxin [24]. Both BL8 cells and cell free supernatant showed cytotoxic activity against Vero cell line (Figure 2). BL8 cells had significantly higher cytotoxicity compared to cell free supernatant, both after 2 hrs and 4 hrs of incubation (p = 0.0066 and p = 0.023 respectively). This suggests that cytotoxicity of *Clostridium* sp. BL8 might be largely due to cell bound factors. A significant difference was observed in the reduction of cell count between 2 hrs and 4 hrs of incubation (p = 0.003), this suggests that cytotoxic activity of *Clostridium* sp. BL8 might be time dependent.

## Conclusion

*Clostridium* sp. BL8 seems to have adapted for survival in the human gut environment, with presence of different adaptive features. *Clostridium* sp. BL8 genome showed presence of an array virulence factors encountered in different pathogens, suggesting that it has a high potential to cause infections in humans. This study thus, lays the basis for further *in vivo* and *in vitro* studies that are necessary to decipher the pathogenesis of infections that can be caused by a novel potential pathogen *Clostridium* sp. BL8 and guide in the treatment of these infections.

## Competing interests

The authors declare that they have no competing interests.

## Authors’ contributions

NPM, SAS, YSS and DR designed the study. NPM, SAS, VL and MHR performed the experiments. NPM and SAS analyzed the data and wrote the manuscript. MHR, VL, YSS and DR checked and edited the manuscript. All the authors have read and approved the manuscript.

## Supplementary Material

Additional file 1: Table S1. The list of all the annotated proteins by RAST server for *Clostridium* sp. BL8 genome. Click here for file
